# Episomal HIV-1 DNA and its relationship to other markers of HIV-1 persistence

**DOI:** 10.1186/s12977-018-0398-1

**Published:** 2018-01-30

**Authors:** Javier Martinez-Picado, Ryan Zurakowski, María José Buzón, Mario Stevenson

**Affiliations:** 1 AIDS Research Institute IrsiCaixa, University Hospital Germans Trias i Pujol, Ctra. de Canyet s/n, Badalona, 08916 Barcelona, Spain; 2 University of Vic-Central University of Catalonia (UVic-UCC), Vic, Spain; 3 Catalan Institution for Research and Advanced Studies (ICREA), Barcelona, Spain; 4 Department of Biomedical Engineering, University of Delaware, Newark, DE USA; 5 Infectious Diseases Department, Vall d’Hebron Research Institute, Hospital Universitari Vall d’Hebron, Barcelona, Spain; 6 Division of Infectious Diseases, Department of Medicine, University of Miami Miller School of Medicine, Miami, FL USA

## Abstract

Reverse transcription of HIV-1 results in the generation of a linear cDNA that serves as the precursor to the integrated provirus. Other classes of extrachromosomal viral cDNA molecules can be found in acutely infected cells including the 1-LTR and 2-LTR circles of viral DNA, also referred as episomal HIV-1 DNA. Circulating CD4^+^ T-cells of treatment-naïve individuals contain significant levels of unintegrated forms of HIV-1 DNA. However, the importance of episomal HIV-1 DNA in the study of viral persistence during antiviral therapy (ART) is debatable. 2-LTR circles are preferentially observed in the effector memory CD4^+^ T cell subset of long-term treated subjects. Treatment intensification of standard regimens has been used to determine if more potent ART can impact viral reservoir activity. Adding a potent antiretroviral drug to a stable triple-drug regimen has no measurable impact on plasma HIV-1 RNA levels, suggesting that ongoing cycles of HIV-1 replication are not a major mechanism driving persistent plasma viremia during triple-drug ART. However, in randomized clinical trials of HIV-1-infected adults on apparently effective ART, the addition of an integrase inhibitor (raltegravir) to stable regimens resulted in a transient increase in 2-LTR circles in some patients, suggesting a pre-intensification steady-state in which the processes of virion generation and de novo infection were occurring. Mathematical modeling of 2-LTR production during integrase inhibitor intensification suggests the coexistence, at different levels, of ongoing de novo infection and de novo replication mechanisms, specifically in inflamed lymphoid drug sanctuaries. Most reports looking into potential changes in 2-LTR circles in interventional clinical studies have simultaneously assessed other potential surrogate markers of viral persistence. Transient increases in 2-LTR circles have been correlated to decreases in CD8^+^ T-cell activation, transient CD45RA^−^CD4^+^ T-cell redistribution, and decreases in the hypercoagulation biomarker D-dimer in ART-intensified individuals. It is difficult, however, to establish a systematic association because the level of correlation with different types of markers differs significantly among studies. In conclusion, despite suppressive ART, a steady-state of de novo infection may persist in some infected individuals and that this may drive immune activation and inflammation changes reflecting residual viral reservoir activity during otherwise apparently suppressive ART.

## The nature of episomal HIV DNA

Integration into host-cell DNA is an essential step in the life cycle of all retroviruses, including HIV-1. Once integrated, the provirus is replicated as an integral element of the host genome, efficiently transcribing viral DNA into new copies of the viral genome and mRNAs that encode viral proteins [1]. Integration also is important to viral persistence. By integrating within host cell DNA, the virus essentially usurps the life span of the infected cell. Therefore, integration within long lived cells such as memory CD4^+^ T cells and macrophages contributes to HIV-1 persistence in the host. Furthermore, during mitosis, proviruses are duplicated in each daughter cell and thus homeostatic proliferation of infected cells provides an additional mechanism for proviral persistence [2–7].

Reverse transcription results in the generation of a linear cDNA that serves as the precursor to the integrated provirus. In addition, other classes of extrachromosomal viral cDNA molecules can be found in acutely infected cells including (1) 1-long terminal repeat (1-LTR) circle, which is most likely the result of homologous recombination between the LTRs of the linear DNA molecule; (2) 2-LTR circles, whose structure is consistent with the ligation of the two ends of the linear precursor, often with deletions or insertions of a few nucleotides at the “circle junction” [1]. The 1-LTR and 2-LTR closed circular DNA are also referred as episomal HIV-1 DNA. However, unlike episomal DNA molecules of herpesviruses such as Epstein Barr Virus (EBV) that contain elements allowing autonomous episomal replication, episomes generated by HIV-1 cannot replicate autonomously.

Estimates of the efficiency with which newly synthesized viral cDNA molecules complete the subsequent steps leading to integration are technically difficult to obtain. However, under favorable in vitro conditions, between 10 and 30% of the viral cDNA molecules synthesized in acutely infected permissive cells will ultimately become integrated [8, 9]. Therefore, unintegrated forms represent the largest fraction of HIV-1 cDNA in the nucleus. The suggested relative abundance is greater for unintegrated linear DNA followed by integrated provirus, 1-LTR circles (~ 10%), and finally 2-LTR circles (~ 1%) [9–11]. Kinetically, they seem to appear in the same order [9]. While the linear molecule is the direct precursor to the integrated provirus, the circular forms appear to be dead-end by-products and do not serve as intermediates in the viral replication cycle. Since the free ends of linear viral DNA mimic double strand breaks of the chromosome and thus may provide a signal for apoptosis, circularization might be considered as a repair process to reduce such cellular danger signals [12]. Interestingly, recent data suggest that 2-LTR circles can also be used as a reserve supply of genomes for proviral integration [13]. However, this hypothesis has only been described in ex vivo experiments and its potential role in the overall HIV-1 replication cycle in vivo remains to be determined.

There are different PCR-based molecular methods for the specific detection and quantification of 2-LTR circles. A recent review has been devoted to precisely compare their properties and limitations [14]. The more recently developed digital droplet PCR (ddPCR) technology [15] is replacing conventional PCR methods [16, 17]. Even if the relative abundance of unintegrated 1-LTR circles has been suggested to be tenfold greater than that of 2-LTR circles [9], methods for quantification of either 1-LTR circles or unintegrated viral linear DNA have only been developed in in vitro experiments [18], but not systematically applied to samples from clinical trials. As HIV-1 lacks necessary factors for the maintenance of episomal replication, it has been argued that these episomes are labile intermediates in the virus life cycle and as such, indicative of recent infection events [19]. However, the lability of 2-LTR circles has been questioned by in vitro experiments in some HIV-1-infected cell lines [20–22]. This specific aspect will be further discussed in the next sections.

It has been suggested that unintegrated HIV-1 DNA molecules can be transiently transcribed and perhaps even support virus production, latency and immune responses, especially during direct infection of resting CD4^+^ T cells [23–25]. Moreover, it has been shown that Nef expressed from extrachromosomal DNA, downregulates CD4 surface expression on primary CD4^+^ T lymphocytes, albeit not as efficiently as integrating virus [25, 26]. It is, however, uncertain whether the accumulation of unintegrated viral DNA might have a role in clinical pathogenesis. It has also been suggested that abundant unintegrated viral DNA is a manifestation of high-multiplicity of infection [27] that could occur during cell-to-cell transmission in lymphoid tissues rather than in peripheral blood. This hypothesis would be compatible with the potential enrichment of 2-LTR circles in CD4^+^ T lymphocytes migrating from tissues to peripheral blood [28, 29].

## Episomal HIV-1 DNA in the study of viral persistence during ART

The existence of high levels of unintegrated HIV DNA in vivo was first demonstrated in blood and brain tissue of AIDS patients, with a maximum ratio of unintegrated to integrated HIV of approximately 80:1 [30]. It was also latter shown that during the asymptomatic phase of infection there was an extremely low total body load of latently infected resting CD4^+^ T cells with replication-competent integrated provirus. In treatment-naïve individuals, the most prevalent form of HIV-1 DNA in resting and activated CD4^+^ T cells is a full-length, linear, unintegrated form that is not replication competent [31].

The implementation after 1996 of combination antiretroviral therapy (ART) resulted in sustained suppression of viral replication in HIV-1-infected subjects. However, it was soon evident that a reservoir of latently infected cells established early in infection maintained viral persistence in the face of suppressive ART [31–33]. In this context, the detection of episomal HIV-1 DNA infection intermediates, specifically 2-LTR circles, in a large percentage of infected individuals on highly active ART, despite sustained undetectable levels of plasma viral RNA, was interpreted as suggesting that cells were still in the process of being infected [19]. Moreover, 2-LTR circles were preferentially observed in the effector memory CD4^+^ T-cell subset compared to naive, memory stem cells, central-memory and terminally differentiated CD4^+^ T cell subsets of long-term treated subjects [34]. This suggests that in the context of potent cART, more differentiated/activated cells are preferentially supporting new rounds of viral infection. The utility of 2-LTRs as indicators of an acute infection was questioned by in vitro experiments in some HIV-1-infected cell lines [20–22]. However, it was unclear whether such short-duration in vitro experiments in cell lines were predictive of episomal dynamics in vivo. To address whether 2-LTR episomes were labile in vivo, Sharkey et al. analyzed the dynamics of episomal cDNA turnover in vivo by following the emergence of an M184 V polymorphism in plasma viral RNA, in episomal cDNA, and in proviral DNA in patients on suboptimal therapies [35]. Acquisition of drug resistance in plasma resulted in a complete replacement of wild-type viral RNA genomes with genomes harboring the M184 V mutation within 2 weeks of treatment initiation. In the majority of individuals, episomes harboring the wild-type M184 codon had been replaced with episomes harboring a V184 codon within 6 weeks of treatment initiation. Importantly, a complete replacement of wild-type episomes with M184 V-containing episomes occurred while proviruses remained wild type over the 52-week duration of the study [35]. This study provided in vivo evidence that 2-LTRs are labile and dynamic. It is possible that the loss of wild-type episomes was due to turnover of the infected cell, but the time-scale of the turnover is faster than could be explained by the natural turnover of resting T-cell populations. Accelerated host cell turnover could be explained by viral cytopathicity and/or host immune mediated clearance. However, there is no evidence to suggest that cells harboring episomes are subject wither to viral cytopathicity or immune surveillance. Either process would require that episomes are sufficiently transcriptionally active to generate enough viral antigen that would promote cytopathicity and serve as an immune target. It is more likely that the rapid turnover observed for episomes reflected the lability of the episome and not the lability of the cell harboring it.

Even after the advent of newer antiretroviral drugs, the size of the latent reservoir in individuals in ART does not seem to change significantly after the first few years of treatment [34, 36, 37]. Therefore, researchers turned their attention on identifying approaches with which to gauge viral activity in individuals on effective ART as well as the impact of more potent antiviral regimens. Given the lack of any direct methods to quantify reservoir activity in vivo, we and others have used treatment intensification of standard regimens to determine if more potent ART can impact viral reservoir activity. With regard to residual plasma HIV-1 RNA, the results of these studies have been very consistent: adding a potent antiretroviral drug to a stable triple-drug regimen has no measurable impact on plasma HIV-1 RNA levels [38–45], suggesting that ongoing cycles of HIV-1 replication are not a major mechanism driving persistent plasma viremia during triple-drug ART.

**Fig. 1:**
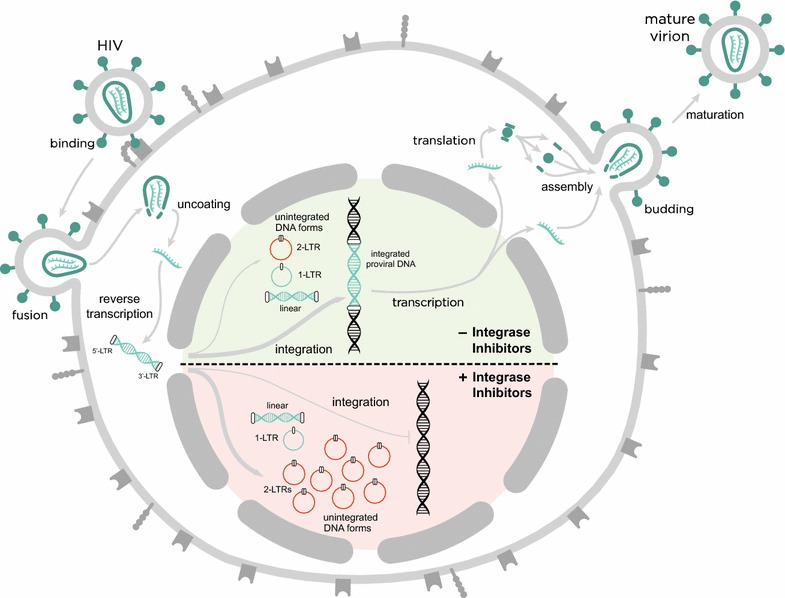
Schematic overview of the HIV-1 replication cycle in presence or absence of integrase inhibitors. The figure illustrates the main steps in the HIV-1 life cycle divided into an early and a late phase. The early phase includes: the attachment of the virus particle to the CD4 receptor and co-receptors CCR5 or CXCR4; the fusion with the host cell membrane; the uncoating of the viral capsid; the release of the HIV-1 RNA genome and proteins into the cytoplasm; the reverse transcription of the viral RNA genome into a DNA duplex, which has terminal duplications known as long terminal repeats (LTRs); the translocation into the nucleus and the integration into the cell genome. In the nucleus, unintegrated viral DNA is found in both linear and circular forms. The unintegrated circular forms of viral DNA have either one or two LTRs. The linear unintegrated viral DNA is the precursor of integrated proviral DNA, which is a stable structure that remains indefinitely in the host-cell genome. After the integration, the late phase of the cycle starts in which: the proviral DNA is transcribed to form new viral RNA, which subsequently is translated to form viral proteins; these proteins translocate to the cell surface to assemble in the cell membrane and form new viruses. Finally, the new viral particles bud off and are released as mature virions. Adapted from [76]

The unique mechanism of action of the integrase inhibitor drug class allowed us, and others, to indirectly assess whether new rounds of infection could occur under suppressive ART. These drugs inhibit the integration of episomal HIV-1 DNA strands—including 2-LTR circles—into the host genome (Fig. 1). In two randomized clinical trials of HIV-1-infected adults on apparently effective ART, the addition of an integrase inhibitor (raltegravir) to a stable regimen resulted in a transient increase in 2-LTR circles. [46, 47]. An increase in episomal DNA with inhibition of integration suggests that at baseline (pre-intensification), there existed a steady-state in which the processes of virion generation and de novo infection were occurring despite the fact that all trial participants were fully suppressed on two reverse transcriptase inhibitors plus a protease inhibitor (PI) for the duration of the study. This phenomenon was observed in approximately 30% of subjects, with most of the activity observed in those who had been on a PI-based regimen. An acute increase in 2-LTR circles requires the presence of infectious virions and reverse transcription of a linear cDNA, which is the precursor for 2-LTR circles. Since virions and linear viral cDNAs are labile, de novo infection is necessary to drive increase in 2-LTRs upon raltegravir intensification. An important distinction needs to be made here. Recently produced infections virions may be produced by one of two pathways, either arising from pre-existing long-lived reservoir cells, or potentially from ongoing cycles of replication. Since either pathway will result in an increase in 2-LTR production following raltegravir intensification, an increase in 2-LTR circles following intensification only proves the existence of de novo infection, not necessarily de novo replication. However, as we will discuss later, the dynamics of 2-LTR following intensification do allow us to distinguish between these two pathways. Although the clinical significance of these events is unclear, it is evident that even under effective ART, there is some replenishment of infected cells by new rounds of infection.

## Mathematical modeling of episomal HIV DNA

The intensification experiments discussed above shared several key characteristics that can guide modeling efforts. The patients had been on stable antiviral therapy with suppressed plasma viremia for extended periods prior to intensification, so it is reasonable to assume that the dynamics were at steady-state prior to the intensification. The administration of integrase inhibitor was sustained for the duration of the experiment, so the perturbation was monotonic (i.e., there were no complicated dynamics introduced by the perturbation itself). Finally, the response in the measured 2-LTR dynamics was characterized by a sharp increase followed by a gradual decrease, with the final concentration of 2-LTR episomes often below the baseline level.

### Modeling 2-LTR production during integrase inhibitor intensification

**Fig. 2:**
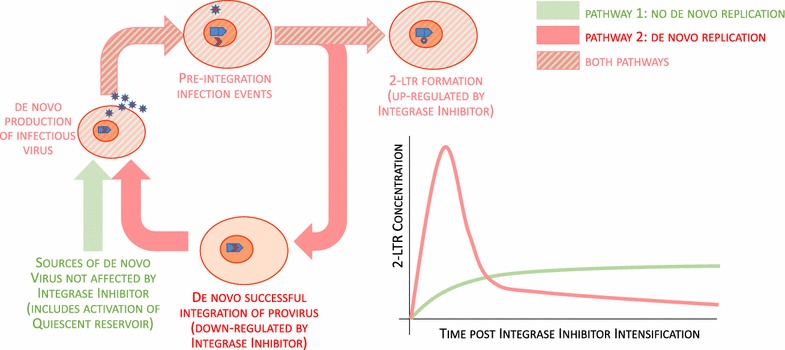
Interactions between integrase inhibitor, uninfected CD4^+^ T cells, infected CD4^+^ T cells, 2-LTR, and HIV virions during treatment with integrase inhibitors. Promotion and suppression pathways are color-coded, showing that the suppression pathway only exists when ongoing replication is assumed

Integrase Inhibitors are known to promote the formation of 2-LTR episomes by blocking the activity of the integration complex, leaving the unintegrated linear HIV DNA as a target for the host DNA repair enzymes. The observed increase in 2-LTR is therefore best explained as the interruption of HIV integration events that would otherwise have occurred in the absence of integrase inhibitor. However, the presence of ongoing integration events does not necessarily imply the presence of ongoing rounds of replication, as there is a known pathway whereby quiescent infected cells may become active, produce virus, and create new infections that proceed to the integration phase without necessarily progressing to the virus-producing stage [48]. This is illustrated as pathway 1 in Fig. 2, which also shows the characteristic response in 2-LTR expected if pathway 1 dominates the source of the de novo infection events [49].

This pathway, however, is not capable of explaining the subsequent observed decrease in 2-LTR, integrase inhibitors have no suppressive effect on any step of this pathway (activation of quiescent infected cells, production of virus, viral entry and reverse transcription, formation of pre-integration complex). If we assume the presence of ongoing rounds of replication prior to intensification, illustrated (together with its characteristic response) in Fig. 2 as pathway 2, then a suppressive pathway exists where integrase inhibitor blocks the successful integration of HIV DNA, suppressing the population of virus-producing cells, suppressing the production of virus, cell entry, and formation of pre-integration complexes, and ultimately suppressing the production of 2-LTR episomes. An exhaustive search of possible monotone effect models [50, 51] has shown that only models that assume ongoing rounds of successful replication have suppressive pathways that are consistent with the known effects of integrase inhibitors [52]. The observed increase in 2-LTR following intensification, together with the subsequent decrease, can only be explained by the presence of successful rounds of HIV replication prior to intensification.

By fitting patient data to the model described above [49], we can determine how much of the virus involved in the de novo infection events was sourced from successful rounds of de novo replication, and how much came from sources unaffected by raltegravir intensification (including quiescent reservoir cells). We are also able to obtain estimates of the amount of viral replication prior to intensification necessary to produce the observed 2-LTR dynamics post-intensification. We have previously reported the simplest quantitative model capable of describing the 2-LTR behavior post-intensification [49]. This model tracked virus-producing infected cells, which may be created either by new infection events or by the activation of quiescent infected cells, and 2-LTR-containing cells.

The relatively sparse number of time points available mean than only the data from the Buzon trial [43, 46] had sufficient power to provide meaningful estimates of these rates, and even these estimates gave broad confidence intervals for most parameters. Interestingly, however, the estimates for the infection success ratios were very tightly constrained by the data, with seven of the patients in the study having this ratio bounded between 99 and 100%. This means that almost all of the integration events occurring in these patients prior to intensification came from ongoing replication (pathway 2), with only a negligible amount coming from the activation of quiescent infected cells (pathway 1). By contrast, the other six patients with positive 2-LTR measurements following intensification did not have a tight constraint on the infection ratio, meaning that pathway 1 and pathway 2 explained the 2-LTR measurements equally well. The data from the Buzon trial [46] therefore provided evidence of at least ongoing de novo infection in 30% of the patients, and evidence of de novo replication in 15% of the patients. The amount of ongoing infection necessary to explain the data, while poorly constrained, was nevertheless quite high, with median estimates on the order of 10^7^–10^8^ infected cells per day in the eight patients described.

### Unchanging plasma viremia implies sanctuary-site origin of 2-LTR

The high success ratios of viral infection found in the seven patients following intensification could not occur in the presence of suppressive levels of antiretrovirals. Furthermore, the levels of ongoing infection required to explain the observed 2-LTR dynamics in the thirteen patients would correspond to measurable level of plasma virus if it were evenly distributed through the body. Since plasma viral loads in the intensification trials remained below the standard limit of detection in all cases, it is necessary to assume that the 2-LTR formation (and the ongoing replication that produced it) are occurring in a site with non-suppressive concentrations of antiretrovirals, sufficiently isolated from the blood that transport of free virus and actively infected cells are greatly reduced, but sufficiently connected to the blood that transport of 2-LTR-containing cells is possible. There is growing experimental evidence that many lymphoid tissues may serve as sanctuary sites [53, 54], with biopsy studies demonstrating non-suppressive levels of antiretrovirals in the lymphoid tissues of apparently suppressed patients. In fact, preliminary viral genetic analysis suggests that cells sequestered in tissues, rather than circulating T cells, might be responsible for supporting virus replication [28, 29].

**Fig. 3:**
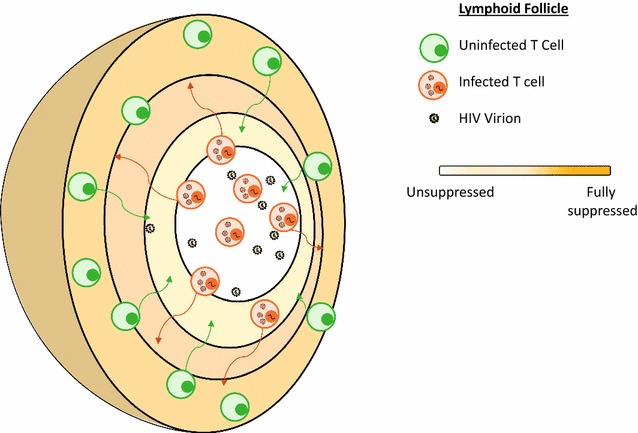
Spatial lymph node model. A true spatial model only allows direct migration between neighboring compartments, which allows for isolation from the blood, and illustrating simultaneously that small compartments cannot sustain replication because of fast transit times

In order to explore the feasibility of ongoing replication within a sanctuary site, we have developed a multi-compartment model of the sanctuary that captures the spatial effects characteristic of a lymphoid tissue-based drug sanctuary (Fig. 3), using infection dynamic rates and tissue transport rates derived from a number of human and animal studies. Analyzing this model leads to several interesting insights [55, 56]. The model demonstrates that a lower limit in sanctuary size is necessary to sustain ongoing replication, even when no antiretrovirals are present. When the site is smaller than this size, target cells and infected cells are more likely to migrate through the site without interacting than remain in the site together long enough to interact. The number of infections occurring in the site under these circumstances is too low to be self-sustaining. A small site might explain increased levels of de novo infection, as per pathway 1, but only a large site could sustain the de novo replication of pathway 2. Based on the estimates used for T-cell transport rates, the minimum diameter for a sanctuary site to sustain ongoing replication is approximately 0.2 mm. This is larger than the average size of a lymphoid follicle, but is consistent with the size of inflamed lymphoid follicles [57]. This shows that inflammation is a necessary condition for active replication in a sanctuary site. We would therefore expect signatures of ongoing replication in the 2-LTR dynamics post-intensification to correlate with markers of systemic inflammation pre-intensification.

When multiple lymphoid sites with diameters larger than 0.2 mm, a total dispersed site volume over 30 mL, and sufficiently low antiviral activity are modeled, we find that ongoing infection rates as high as 10^7^–10^8^ infected cells per day are consistent with no measurable change in the plasma virus level. When the addition of integrase inhibitor to these sites is simulated, the dynamics of 2-LTR formation in the site and their migration to the plasma produce curves identical to those seen in the data from the integrase inhibitor intensification trials. The mathematical models demonstrate that ongoing replication in inflamed lymphoid drug sanctuaries is a feasible explanation for the 2-LTR dynamics observed in seven patients following intensification. Recent data also suggest that HIV-1 actively evolves within lymph nodes due to low antiretroviral drug penetration in patients with undetectable levels of virus in their bloodstream [58], highlighting how dynamic and spatial processes act together to permit HIV-1 to persist within the infected host.

### Observed half-life of 2-LTR in the PBMCs is consistent with activated T-cell turnover

The stability of 2-LTR episomes in vivo has been a subject of some controversy [19–22, 59, 60]. In vitro assays have shown the 2-LTR to be stable on short time scales, but in vivo studies have demonstrated 2-LTR dynamics consistent with a high turnover rate. In the model of sanctuary site dynamics described above, the 2-LTR in the intensification trial are formed in T cells in an environment of high inflammation, which will likely result in a high rate of T-cell activation and proliferation. The vast majority of activated T-cells during a proliferative immune response have short lifespans, with only a minority surviving to revert to long-lived memory cells [61]. The estimated half-life of the 2-LTR circles from these studies, when bias-corrected for the fact that measurements were made in the blood, not the sanctuary site [62], is fully consistent with the turnover rate of uninfected, activated, proliferating T-cells as estimated from patients with uncontrolled HIV infection [63, 64]. The observed 2-LTR turnover in this experiment can easily be explained either by native lability of the circles, or by the turnover rate of the host cells.

## Relationship to other markers of viral persistence

One of the major problems for the research community that investigates HIV-1 persistence in subjects on suppressive ART (< 50 copies HIV-1 RNA/ml) is the precise quantification of viral reservoirs and potential residual levels of viral reservoir activity. The complexity is threefold: first, the very limited size of viral reservoirs that are sufficient to reset the infection if ART is withdrawn. Second, the multiplicity of assays to measure viral persistence; these range from replication competent virus to the simple presence of proviral DNA. While proviral DNA is a convenient and easy way to measure moiety (by for example droplet digital PCR), the majority of proviruses are archival and non-functional, and not likely to inform on the dynamic nature of the viral reservoir. Cell-associated viral RNA, cell-associated viral proteins, or inducible virus have also been used as surrogates for viral reservoir activity. Individually, these measurements do not provide comprehensive insight into viral reservoir size or activity and as such, multiple surrogates are normally included in studies of viral persistence. Because HIV-1 infection is inevitably linked to increases in cellular activation and inflammation, other immune-based markers are systematically considered, even in the settings of controlled plasma viremia, including levels of T-cell activation in total CD4^+^ T cells or specific subpopulations, as well as plasma biomarkers of inflammation (CRP, IL-6), coagulation (D-Dimer) and bacterial translocation (sCD14). And third, the challenge in understanding viral persistence in peripheral blood and tissues, especially lymphoid-associated tissues where the majority of the principal cell target for HIV-1, the CD4^+^ T lymphocytes, reside.

As a consequence, most of the reports looking into potential changes in 2-LTR circles in interventional clinical studies have simultaneously assessed other potential surrogate markers of viral persistence. It is difficult, however, to establish a systematic association between them, because the type of markers differs significantly among studies.

Our first prospective, open-label, randomized study comprising 69 individuals on suppressive triple-drug antiretroviral treatment that were randomly assigned 2:1 to receive raltegravir intensification over 48 weeks, resulted in a specific and transient increase in episomal DNAs in approximately 30% of the study participants. In subjects exhibiting an elevation in episomal DNAs, immune activation was higher at baseline and was subsequently normalized after raltegravir intensification [43, 46]. Subsequent raltegravir discontinuation for 12 weeks resulted in a partial rebound in CD8 activation markers in the 2-LTR+ subgroup, restoring the differences between subgroups observed at study entry, particularly in terms of CD38 expression within the CD8 memory T-cell compartment [65]. An association was also found between reductions in immune activation and plasma levels of D-dimer (a coagulation biomarker that is predictive of morbidity and mortality among patients receiving treatment for HIV-1 infection), which exclusively decreased in intensified patients on PI-based ART regimens, highlighting the link between ART composition and residual de novo viral infection [66]. Conversely, the perturbation in 2-LTR circles during raltegravir intensification was not associated with detectable changes in total or integrated HIV-1 DNA in peripheral PBMCs, residual plasma viremia, CD4^+^ and CD8^+^ T-cell populations in blood, nor with multiple biomarkers of inflammation in plasma, including sCD14 [43, 46, 66].

A similarly designed study included 31 subjects on suppressive triple-drug antiretroviral treatment. Raltegravir intensification for 24 weeks also resulted in a transient increase in 2-LTR circles, which was also associated with a slight but significant decrease in the D-dimer level in plasma [47]. No differences were however found for residual plasma viremia, total HIV DNA in peripheral PBMCs, CD4^+^ and CD8^+^ T-cell populations or their activation levels in blood, as neither in IL-6, a biomarker of inflammation in plasma [47].

Alternative studies have shown no effect of raltegravir intensification on viral replication markers in the blood of HIV-1-infected patients receiving ART, including 2-LTR circles [40]. Nevertheless, the transient increase in 2-LTR circles in this study might have been missed as the first sample was taken 12 weeks after intensification. In any case, 12 out of 50 subjects had detectable 2-LTR circles at baseline, in association with higher residual plasma viremia and higher total HIV-1 DNA in CD4^+^ T cells [40]. Similarly, nine chronically HIV-1-infected subjects on stable ART intensified their therapy with raltegravir in a non-comparative pilot trial, with no significant short-term effect on a qualitative measure of 2-LTR circles [45].

Another prospective, open-label, randomized study, comprising 30 individuals, evaluated the effect of adding maraviroc to a raltegravir-based regimen in early HIV-1 infection for 24 weeks [67]. There was again a transient increase in 2-LTR circles in both groups early after initiation of treatment, which decreased earlier in maraviroc-treated individuals. Residual plasma viremia, absolute CD4^+^ T-cell and CD8^+^ T-cell counts, immune activation, CD4^+^/CD8^+^ T-cell ratio, and soluble inflammation biomarkers (D-dimer, CRP, sCD14, Lp-PLA2, sVCAM-1, and IL-6) were similar in both arms at the end of the study [67].

Early studies in HIV-infected individuals who initiated ART reported conflicting information. While one study suggested that the 2-LTR circles in a subgroup of patients rapidly decayed following initiation of suppressive ART [68], other suggested a low impact of ART on 2-LTR circle levels in vivo despite significant changes in plasma viremia and infectious cell frequency [59]. Short-term antiretroviral treatment interruptions showed a profound correlation between levels of total HIV-1 DNA and unintegrated episomal DNA, but treatment discontinuation for only 15 days, followed by treatment resumption, did not alter any of these two parameters [69].

Outside of the context of interventional clinical trials, the level of 2-LTR circles has been compared with other measures of viral reservoirs in HIV-1 eradication studies. Detection and quantitation of 2-LTR circular DNA was shown to correlate with the frequency of the recovery of replication-competent virus from PBMC obtained from effectively treated patients [70]. More recently, 2-LTR circles in PBMCs or resting CD4^+^ T cells were shown not to correlate with infected cell frequencies measured in the viral outgrowth assay either in acute or chronically infected subjects [71]. The geometric mean level of 2-LTR circles was 27-fold and 34-fold lower than the total level of HIV-1 DNA measured in PBMC and purified resting CD4^+^ T cells respectively [71]. Another report found lower levels of episomal 2-LTR circles in peripheral PBMCs of ART-treated patients (either early infected or chronically infected) and LTNPs, compared to recent ART-naïve seroconverters [72]. However, this study found no correlation between 2-LTR circles and HIV-1 usRNA, total or integrated HIV-1 DNA, or the CD4/CD8 ratio. It has been also found that 2-LTR circles are much more common in the PBMC of elite suppressors compared to other HIV-1 infected individuals, either treated or untreated [73]. Coincidentally, elite suppressors in this study had lower levels of integrated HIV-1 DNA than other HIV-1 infected individuals, which has also been observed in later reports [34]. However, no formal correlation between the levels of 2-LTR circles and integrated HIV-1 DNA was attempted [73].

These results suggest that, despite suppressive ART, a steady state of de novo infection may persist in some infected individuals and that this may drive immune activation. The decrease in CD8^+^ T-cell activation, a transient CD45RA^−^ CD4^+^ T-cell redistribution, and decreases in the hypercoagulation biomarker D-dimer in intensified individuals may reflect residual viral reservoir activity during otherwise apparently suppressive ART.

## Clinical implications

There is still significant controversy regarding to what extent either single or multiple markers of viral persistence can provide clinically meaningful information. Suggested interventions to reduce the level of viral reservoirs, residual viral replication or low-level viral replication do not necessarily translate into actual clinical benefit.

In the context of theoretically suppressive HIV-1 therapies, antiretroviral regimens based on PI might be less able to achieve the same level of viral suppression as suggested by the more frequent transient increase in 2-LTR circles when triple-drug therapies are intensified with raltegravir [46, 47]. However, the sole virologic success rates of subjects with continued virologic suppression under triple-drug suppressive regimens based on PI does not seem to be different from patients on other regimens.

Raltegravir, as the first clinically available integrase inhibitor, has been exhaustively explored in its potential to contribute to HIV-1 cure strategies. Elvitegravir and dolutegravir are running a little bit behind in such exploratory studies. Newer, still in-development antiretroviral drugs will potentially add novel possibilities to the design of HIV-1 eradication strategies. Such strategies will have to prove their efficacy not only in peripheral blood but also in multiple lymphoid tissues throughout the body where the vast majority of target cells for HIV-1 reside. In this regard, new methodologies that could allow the quantification of markers of viral persistence in intact lymphoid cells obtained from less accessible tissues might play a pivotal role [74, 75]. In fact, the specific detection of 2-LTR circles in tissue-resident lymphoid cells has not yet been reported, either in a descriptive way or in the setting of an interventional strategy to reduce viral persistence in otherwise well-treated individuals.
